# Eight weeks of mineralocorticoid blockade does not improve insulin sensitivity in type 2 diabetes

**DOI:** 10.14814/phy2.14971

**Published:** 2021-08-04

**Authors:** Stine H. Finsen, Mie R. Hansen, Joachim Hoffmann‐Petersen, Henrik F. Højgaard, Stefan P. Mortensen

**Affiliations:** ^1^ Department of Cardiovascular and Renal Research University of Southern Denmark Odense Denmark; ^2^ Department of anesthesiology Odense University Hospital Svendborg Denmark; ^3^ Department of anesthesiology Odense University Hospital Odense Denmark

**Keywords:** aldosterone, insulin sensitivity, mineralocorticoid blockade, type 2 diabetes

## Abstract

Individuals with type 2 diabetes have an increased risk of cardiovascular disease. A correlation between plasma aldosterone and hyperinsulinemia has been demonstrated in vivo, and hyperinsulinemia and insulin resistance are independently associated with the development of cardiovascular complications. We investigated if mineralocorticoid blockade (Eplerenone) improves insulin sensitivity in individuals with type 2 diabetes compared to healthy controls. We included 13 participants with type 2 diabetes (<5 years; male/female, Caucasians) and 10 healthy control participants (male/female, Caucasians). On 2 experimental days, before and at the end of the 8 weeks of treatment with mineralocorticoid blockade, a two‐stage hyperinsulinemic‐isoglycemic clamp (20 and 50 mU∙m^−2^min^−1^) was performed for the determination of insulin sensitivity. No change in insulin sensitivity was detected at the end of the mineralocorticoid blockade in the individuals with type 2 diabetes or the healthy controls. Both before and at the end of the treatment with mineralocorticoid blockade, the individuals with type 2 diabetes had a lower insulin sensitivity compared to healthy controls. In conclusion, mineralocorticoid receptor blockade does not appear to improve insulin sensitivity in individuals with type 2 diabetes.

**Clinical Trial Registration:**

NCT03017703. https://clinicaltrials.gov/ct2/show/NCT03017703

## Graphical abstract

Individuals with type 2 diabetes have an increased risk of developing cardiovascular complications and a correlation between plasma aldosterone and hyperinsulinemia has been shown in vivo. Despite this, the present study does not show a beneficial effect of blockade of the mineralocorticoid receptor in individuals with type 2 diabetes compared to healthy controls.

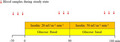

**New & Noteworthy**

The effect of 8 weeks of mineralocorticoid blockade was examined, as a mono‐treatment, in individuals with type 2 diabetes and compared to healthy controls. A two‐stage hyperinsulinemic‐isoglycemic clamp for quantitatively measurement of insulin sensitivity of the skeletal muscle was performed before and after the treatment. The treatment showed no beneficial effect on insulin sensitivity in individuals with type 2 diabetes.

## 1 INTRODUCTION

Individuals with primary hyperaldosteronism have been shown to present with impaired glucose tolerance (Hitomi et al., 2007) in addition to, an increased risk for the development of insulin resistance, leading to type 2 diabetes (Adler et al., 2020; Garg et al., 2010). Besides angiotensin II being the main mediator of aldosterone synthesis in the zona glomeruloza of the adrenal cortex (Cannavo et al., 2018; Pereira et al., 2014), insulin is able to stimulate the synthesis of aldosterone in the zona glomerulosa cells, shown by in vitro experiments (Briet & Schiffrin, 2011). Correlated with these results, in vivo studies have showed a relationship between hyperinsulinemia and levels of plasma aldosterone (Briet & Schiffrin, 2011).

Besides the action of insulin on glucose metabolism, insulin plays an important role on the vascular homeostasis (Bender et al., 2013; Cardillo et al., 1999; Muniyappa & Sowers, 2013). Upon binding to the insulin receptor, insulin receptor substrate‐1 (IRS‐1) activation initiates the phosphatidylinositol 3‐kinase (PI3K) signaling pathway in the endothelial cells (Cardillo et al., 1999), which generates the formation of nitric oxide (NO) (Jansson, 2007; Muniyappa & Sowers, 2013). In the vascular smooth muscle cells (VSMC), a decrease in the intracellular [Ca^2+^] occurs by activation of the IRS/PI3K‐pathway (Bender et al., 2013). Opposing the vasodilatory response, mitogen‐activated protein kinase (MAPK) is additionally activated by insulin, which promotes synthesis of the vasoconstrictor endothelin‐1 (ET‐1) in the endothelial cells (Cardillo et al., 1999; Jansson, 2007; Muniyappa & Sowers, 2013). In the healthy vasculature, vasodilation is dominant, resulting in an increase in blood flow and thereby enhancing glucose delivery in the skeletal muscle (Kim et al., 2006; Muniyappa & Sowers, 2013).

During the continuous hyperinsulinemic state in insulin resistance, a suggested impairment of the IRS‐1/PI3K pathway might occur, diminishing the release of NO and amplifying the MAPK/ET‐1 pathway, causing a shift in the vascular actions of insulin, whereby the vasodilatory response to insulin could be lost (Bender et al., 2013; Muniyappa & Sowers, 2013). These vascular changes, in response to insulin, has been proposed to contribute to increased oxidative states, vascular alterations, and eventually endothelial dysfunction (Bender et al., 2013; Muniyappa & Sowers, 2013). This is supported by an association between an increased risk of cardiovascular events and insulin resistance (Bruder‐Nascimento et al., 2014; Catena et al., 2006; Luther, 2014).

Aldosterone has previously been linked with a pathophysiological role, promoting inflammation, proliferation, and vascular remodeling (Belden et al., 2017; Cannavo et al., 2018; Hermidorff et al., 2017), in part by generation of oxidative stress through formation of reactive oxygen species (ROS) (Paneni et al., 2013). By possible ROS‐dependent mechanisms, a shift in the balance between a vasodilator and vasoconstrictor response to aldosterone in individuals with type 2 diabetes (Finsen et al., 2020) could additionally contribute to cardiovascular disease in this population (Bender et al., 2013; Kenny & Abel, 2019). Previously in vitro studies have shown that aldosterone‐induced ROS additionally are able to cause IRS‐1 degradation, upon binding of aldosterone to the mineralocorticoid receptor (MR) in the VSMC (Hitomi et al., 2007; Hwang et al., 2015). Supporting this, a previous study have demonstrated alterations of insulin secretion in relation to excess aldosterone levels in animals and in murine islets (Luther et al., 2011), as well as plasma aldosterone levels could predict the development of insulin resistance, shown in a previous prospective study (Kumagai et al., 2011).

Individuals with type 2 diabetes have an increased risk for the development of cardiovascular disease compared to individuals with normal insulin sensitivity (Bender et al., 2013). Moreover, hyperinsulinemia and insulin resistance has independently been linked with the increased risk of cardiovascular complications (Bruder‐Nascimento et al., 2014; Catena et al., 2006; Luther, 2014). This indicates that during disease states, such as type 2 diabetes, elevated plasma aldosterone is present, which can result in increased ROS production, endothelial dysfunction as well as increased insulin resistance (Bender et al., 2013; Bruder‐Nascimento et al., 2014; Silva et al., 2015). Collectively, these changes could contribute to vascular alterations (Bender et al., 2013; Bruder‐Nascimento et al., 2014; Silva et al., 2015). Beneficial effects of MR blockade have been suggested to restore the vascular response to insulin and improve insulin sensitivity (Bender et al., 2013; Hwang et al., 2015). To the best of our knowledge, the MR‐dependent effects on vascular insulin signaling in individuals with type 2 diabetes, has yet to be elucidated. The aim of this project was to investigate if 8 weeks of treatment with a MR antagonist, would improve insulin sensitivity in individuals with type 2 diabetes compared to healthy controls. We hypothesized that MR blockade would improve insulin sensitivity in individuals with type 2 diabetes. To address this, we performed a two‐stage hyperinsulinemic‐isoglycemic clamp with measurements of plasma insulin, plasma glucose, and plasma C‐peptide during steady‐state conditions. After an 8‐week period of MR blockade, measurements were repeated to detect any differences between and within groups.

## 2 METHODS

### 2.1 Participants

Thirteen type 2 diabetes participants (<5 years since diagnosis) (male/female Caucasians; 8/5) and 10 healthy control participants (CON) (male/female Caucasians; 5/5) were enrolled. The participants were pseudo‐matched on age and activity level (self‐reported reported activity level). Exclusion criteria included treatment with exogenous insulin, medical blockade of the renin–angiotensin–aldosterone system, hypertension (>140/90 mm Hg), retinopathy, nephropathy, and/orneuropathy of diabetic origin, BMI >32 kg [m2]^−1^, performance of exercise more than 2 h a week, excessive alcohol intake, smoking, or demonstration of liver, cardiovascular and/or renal disease. Prior to inclusion, all participants had a standardized medical examination, including blood pressure measurement (OMRON M3, Comfort), a resting 12‐lead electrocardiography (MAC800 GE Medical systems), and a fasting blood screening including HbA1c, glucose, lipids and markers of renal, hematology, thyroid, and hepatic function. The present data were collected as part of a larger study (Finsen et al., 2020). All included individuals with type 2 diabetes were in stable treatment prior to enrollment, and all included participants were instructed not to refrain from or changed their usual prescribed medication, up to or during the trial. The premenopausal women completed the experimental days at the same time point during their menstrual cycle. The study was approved by the Ethics Committee of Copenhagen and Region of Southern Denmark (H‐15007940) and conducted in accordance with the guidelines of the *Declaration of Helsinki*. The study was registered at *ClinicalTrials.gov* (NCT03017703; “part 2”). Verbal and written informed consents were obtained from all participants before enrollment.

### 2.2 Study protocol

The participants completed 2 experimental days, one before and one at the end of the 8 weeks of treatment with MR blockade (Eplerenone 25–50 mg, Teva Denmark A/S, Kgs.). All participants refrained from caffeine, alcohol, and exercise 24 h prior to each experimental day. Each participant arrived at the laboratory at 8.30 a.m. after an overnight fast (≥ 8 h) and rested in the supine position for the complete trial. After 15 min of rest an arterial cannula (20G, 1.0 × x45 mm with Floswitch, Becton Dickinson Infusion Therapy Systems Inc.) was placed in the radial artery for blood sampling, and a antecubital venous catheter (18 G, 1.3 × 45 mm, luer lock, Mediq Danmark A/S) was placed in the contralateral arm, for infusions of insulin and glucose. After additionally 30 min of rest, baseline blood samples were collected for the determination of fasting plasma glucose, C‐peptide, and insulin.

### 2.3 Hyperinsulinemic‐isoglycemic clamp

**FIGURE 1:**
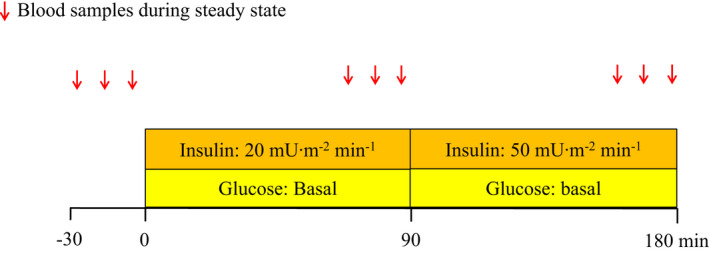
Experimental protocol. A two‐stage hyperinsulinemic‐isoglycemic clamp was performed. At −30, −20 and −10 min before initiating the first stage and during periods of steady state in both clamp stage one and two, blood samples were measured for every 10 min. Red arrows indicate blood samples drawn at steady state periods during baseline, clamp stage one and clamp stage two

Following collection of baseline blood samples, the first stage of the two‐stage hyperinsulinemic‐isoglycemic clamp was initiated with infusion of insulin (Actrapid ®, Humulin R, Novo Nordisk) at a rate of 20 mU∙m^2^ min^−1^. Following 5 min of insulin infusion, a parallel intravenous 20% glucose infusion was started. After 1.5 h of insulin infusion, the second stage of the hyperinsulinemic‐isoglycemic clamp was initiated, increasing the rate of infused insulin to 50 mU∙m^2^ min^−1^, as to ensure complete suppression of the endogenous glucose production. Throughout the first hour of each clamp stage, plasma glucose was measured every 5 min using an ABL835 analyzer (Radiometer Copenhagen), and the infusion of glucose was adjusted according to the results, in order to maintain the baseline fasting plasma glucose level of each participant, creating an individual isoglycemic state. In addition, simultaneous measurements of plasma potassium levels were performed (ABL835), ensuring normal levels. Reaching steady‐state conditions, following the initial hour in each clamp stage, plasma samples for the determination of glucose and potassium levels, in addition to arterial EDTA‐blood samples for later determination of plasma glucose, insulin, and C‐peptide, were collected every 10 min, on three consecutive samples (Figure 1). Steady‐state conditions were determined by a stable blood glucose (≤0.3 mmol L^−1^ glucose between the steady‐state samples) through three consecutives blood samples parallel to an unchanged glucose infusion rate (GIR), interpreting the glucose disposal rate as equal to GIR (Muniyappa et al., 2008). Creating a hyperinsulinemic state increases the risk of hypokalemia and thus, potassium chloride (one tablet; 750 mg Kaleorid^®^, Karo Pharma) was consequently given in the beginning of each experimental day for prevention.

### 2.4 Assays

Blood samples were immediately (<5 min) analyzed for blood glucose and potassium levels (ABL835 analyzer, Radiometer Copenhagen). Arterial EDTA‐blood were centrifuged <15 min at 4000 rpm (4°C) for 10 min. Plasma aliquots were frozen at –80°C until assayed for insulin, glucose, and C‐peptide. Plasma insulin was measured by ELISA (Mercodia AB, Cat# 10‐1113‐01, RRID:AB_2877672), run in duplicates, following the instructions of the manufacture. Plasma glucose was measured using Cobas8000 (Roche Diagnostics, Rotkreuz ZG) following the instructions of the manufacturer, including assessment of hemoglobin (H), lipedema (L), and icterus (I) (HIL‐indices). C‐peptide was analyzed batch‐wise by electro‐chemiluminescence immunoassay (ECLIA) using Cobas e411 analyzer (Roche Diagnostics, Rotkreuz ZG, Schweizerland) with an inter‐ and intra‐assay coefficient variation of <3.8% (QC level 1; 666 pmol L^−1^) and <3.7% (QC level 2; 1544 pmol L^−1^), respectively. Reference range was 400–1600 pmol L^−1^. Baseline levels af plasma aldosterone were measured before initiation of MR blockade, as previously described (Finsen et al., 2020).

### 2.5 Calculations

The individual insulin infusion rate was calculated per body surface area (BSA). BSA was calculated by √((weight (kg)**·**height (cm)) **·** 3600^−1^). Baseline insulin sensitivity was assessed by calculation of the homeostasis model assessment (HOMA2) of insulin resistance (IR) with estimated β‐cell function ([HOMA2]‐%B) and insulin sensitivity ([HOMA2]‐%S), using the computer‐based HOMA2 v.2.2.3 calculator (available at https://www.dtu.ox.ac.uk, released online by the Diabetes Trial Unit) (Mojiminiyi & Abdella, 2010; Wallace et al., 2004). C‐peptide was used to calculate (HOMA2)‐%B and insulin was used to calculate (HOMA2)‐%S. To determine baseline levels of insulin and C‐peptide, a mean of three samples taken at 5‐min intervals were used to compute HOMA2, with a reported intrasubject coefficients of variation of 5.8% for (HOMA2)‐%S and 4.4% for (HOMA2)‐%B, respectively (Wallace et al., 2004). Plasma insulin was used to calculate insulin resistance (HOMA2‐IR).

### 2.6 Mineralocorticoid receptor antagonist

Subsequent to the first experimental day, all participants initiated with 25 mg of Eplerenone per day for the initial 2 weeks, followed by an increase to 50 mg per day up and including the last experimental day. Due to the known adverse side effects of Eplerenone; hyperkalemia and the risk of renal impairment, plasma potassium, creatinine, and carbamide were measured within the first week following initiation and within 1 week after any change of dose. As Eplerenone has a blood pressure lowering effect, each participant performed home blood pressure measurements during the intervention period (OMRON M3, comfort). This was performed daily within the first week after initiation of treatment with the MR blockade, and again daily for 1 week after any change of dose. In addition, blood pressure was measured once a week during the additional 6 weeks of the intervention.

### 2.7 Statistics

Comparison of between‐group baseline characteristics was performed using an unpaired *t*‐test with Welch's correction. To detect any differences within groups, an one‐way repeated measure ANOVA using Tukey's multiple comparisons test, was used. Differences between groups were tested by a two‐way repeated measure ANOVA, with Sidak's multiple comparison post hoc test, to examine any difference between means. Variables of GIR were compared using a two‐tailed Student's *t*‐test, unpaired and paired when appropriate. A D'Agostino & Pearson normally distribution test was conducted to verify the normality assumption, and log transformation was performed if required. If not found normally distributed, despite log transformation, data were analyzed by nonparametric analysis, followed by Dunn's multiple comparison post hoc test. *P*‐values <0.05 were considered significant and a tendency was noted when 0.10 ≤ *p* < 0.05. Statistical analysis was conducted by GraphPad Prism (GraphPad Prism version 8.4.3 for Windows, GraphPad Software, www.graphpad.com). Due to technical reasons all data could not be collected either before or after intervention with MR blockade in three participants (CON *n* = 2, T2D *n* = 1).

## 3 RESULTS

### 3.1 Baseline characteristics of the participants

Baseline characteristics before and following intervention with MR blockade, are presented in Table 1. At baseline, a difference in weight (kg) and BMI was detected between groups. Both before and following the MR blockade, the individuals with type 2 diabetes presented with higher fasting blood glucose and HbA1c compared to healthy controls. In addition, both total cholesterol and LDL cholesterol were lower in the individuals with type 2 diabetes compared to healthy controls, before and following intervention with MR blockade. No difference was found in baseline values of plasma potassium or variables of kidney function, before or following the intervention with MR blockade. A decrease in systolic and diastolic blood pressure was detected in the healthy controls following the intervention with MR blockade, whereas systolic blood pressure tended to be lower in the individuals with type 2 diabetes (*p *= 0.05). No difference in levels of baseline plasma aldosterone between the individuals with type 2 diabetes and the control group was detected before initiation of MR blockade, as previously published (Finsen et al., 2020).

**TABLE 1:** Baseline characteristics of the participants

Variable	Controls		Type 2 Diabetes		*P*	*P*	*P*
	Pre	Post	Pre	Post	Pre‐CON versus T2D	Post‐CON versus T2D	Pre versus post
Participants (*n*)	10	9	13	12			
Male (*n*)	5	4	8	8			
Female (*n*)	5	5	5	4			
Age	52 ± 10		55 ± 6		0.43		
Time since diagnosis	—		2.5 ± 1.2				
Weight (kg)	79.6 ± 15.5	80.1 ± 15.3	92.3 ± 7.3	92.2 ± 6.5	0.02	0.01	
Height (m)	1.74 ± 10.3		1.76 ± 7.4				
BMI (kg [m^2^]^−1^)	26 ± 3		30 ± 2		0.009	0.005	
Total cholesterol	5.0 ± 0.9	4.7 ± 0.8	4.2 ± 0.7	3.8 ± 0.8	0.01	0.02	0.03*/0.0004**
HDL cholesterol	1.6 ± 0.5	1.4 ± 0.3	1.3 ± 0.3	1.3 ± 0.3	0.08	0.41	0.49*/0.01**
LDL cholesterol	2.9 ± 0.8	2.8 ± 0.9	2.1 ± 0.5	1.9 ± 0.7	0.01	0.02	0.04*/0.01**
Triglycerides	1.13 ± 0.56	1.13 ± 0.61	1.45 ± 0.49	1.35 ± 0.60	0.05	0.44	0.40*/0.72**
HbA1c (mmol mol^−1^)	34 ± 3	35 ± 3	53 ± 13	51 ± 10	<0.0001	0.0004	0.22*/0.52**
HbA1c (%)	5.3 ± 0.3	5.4 ± 0.3	7.0 ± 1.2	6.8 ± 0.9	0.0005	<0.0001	0.28*/0.47**
Plasma glucose (mean; mmol L^−1^)	5.8 ± 0.5	5.9 ± 0.5	8.5 ± 1.8	8.2 ± 1.4	0.0004	0.0008	0.46*/0.50**
Potassium (mmol L^−1^)	3.9 ± 0.3	3.9 ± 0.3	4.2 ± 0.4	4.3 ± 0.5	0.11	0.10	>0.99*/0.48**
Creatinine (μmol L^−1^)	80 ± 13	80 ± 15	75 ± 15	72 ± 19	0.38	0.33	0.94*/0.26**
Carbamide (mmol L^−1^)	4.8 ± 1.1	5.9 ± 2.2	4.9 ± 1.6	5.2 ± 1.4	0.87	0.38	0.09*/0.66**
eGFR (ml min^−1^ [1.73 m^2^]^−1^)	82 ± 7	83 ± 10	84 ± 9	85 ± 8	0.45	0.62	0.76*/0.83**
Systolic blood pressure (mm Hg)	129 ± 10	122 ± 12	129 ± 11	125 ± 10	0.99	0.44	0.03*/0.05**
Diastolic blood pressure (mm Hg)	85 ± 8	80 ± 10	84 ± 6	83 ± 7	0.83	0.29	0.02*/0.74**
Glucose lowering medication (*n*)							
Diet	—		1				
Metformin	—		9				
Liraglutide	—		1				
SGL2‐inhibitor	—		1				

Abbreviations: CON, controls; eGFR, estimated glomerular filtration rate; Post, following mineralocorticoid receptor blockade; Pre, prior mineralocorticoid receptor blockade; SGLT2‐inhibitor, selective sodium‐glucose cotransporter 2 inhibitor; T2D, type 2 diabetes.

* *p*‐value in the control group

** *p*‐value in the T2D group.

### 3.2 Effect of MR blockade on glucose turnover

**FIGURE 2:**
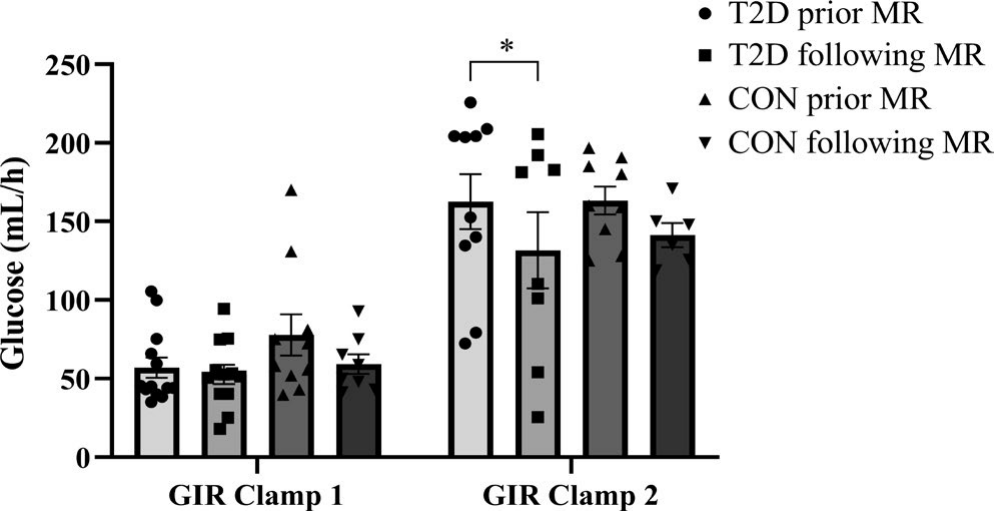
Glucose infusion rate (GIR; mL h‐1) before and following mineralocorticoid blockade (MR). T2D: individuals with type 2 diabetes. CON: control participants. *p = 0.04 in the individuals with type 2 diabetes

Plasma glucose concentrations measured during clamp stage 1 and clamp stage 2, were equivalent before compared to after MR blockade in the individual groups. No difference was detected in insulin levels and insulin resistance, between the individuals with type 2 diabetes and the healthy controls, before compared to after MR blockade. GIR was similar during both clamp stage 1 and clamp stage 2, respectively, before compared to after MR blockade, between the individuals with type 2 diabetes and the healthy controls. Following MR blockade, GIR was equivalent in the healthy controls, compared to before MR blockade. In the individuals with type 2 diabetes, a decrease in the measured insulin sensitivity was detected following MR blockade, during clamp stage 2, compared to before MR blockade (T2D: 153 ± 18 vs. 132 ± 24 ml h^−1^ prior vs. following intervention, *p *= 0.04) (Figure 2). No difference was detected in the achieved insulin response in the individuals with type 2 diabetes, during clamp stage 2 following MR blockade, compared to before MR blockade, and there was no difference in the C‐peptide response in the individuals with type 2 diabetes (Table 2).

**TABLE 2:** Fasting blood glucose (mmol L^−1^), plasma insulin (pmol L^−1^), and plasma C‐peptide (pmol L^−1^) at baseline and during the two‐stage hyperinsulinemic‐isoglycemic clamps

Prior MR blockade						Following MR blockade					
Baseline		Clamp stage 1		Clamp stage 2		Baseline		Clamp stage 1		Clamp stage 2	
T2D	Control	T2D	Control	T2D	Control	T2D	Control	T2D	Control	T2D	Control
**Fasting blood glucose (mmol L^−1^)**											
7.1 ± 0.5	5.4 ± 0.1*	7.5 ± 0.4	5.2 ± 0.1*	7.3 ± 0.5	5.2 ± 0.3*	7.2 ± 0.5	5.1 ± 0.2*	7.5 ± 0.6	5.1 ± 0.3*	6.7 ± 0.4	5.0 ± 0.2*
**Plasma insulin (pmol L^−1^)**											
51 ± 7	34 ± 6	241 ± 19	220 ± 14	536 ± 37	505 ± 36	56 ± 14	29 ± 4	283 ± 30	185 ± 12**	691 ± 102	506 ± 37
**Plasma C‐peptide (pmol L^−1^)**											
0.9 ± 0.1	0.7 ± 0.1*	0.8 ± 0.1	0.5 ± 0.1*	0.7 ± 0.1	0.4 ± 0.1	1.0 ± 0.1	0.69 ± 0.1*	0.9 ± 0.1	0.6 ± 0.1	0.8 ± 0.2	0.8 ± 0.0

Values are presented as mean ± SEM for *n *= 10–13 participants.

Abbreviations: MR, mineralocorticoid receptor; T2D, individuals with type 2 diabetes.

* *p* < 0.05 between individuals with type 2 diabetes and the healthy controls.

** *p* < 0.05 prior versus following intervention between groups.

*** *p* < 0.05 prior versus following intervention in the individual group.

### 3.3 Effect of MR blockade on insulin metabolism

**FIGURE 3:**
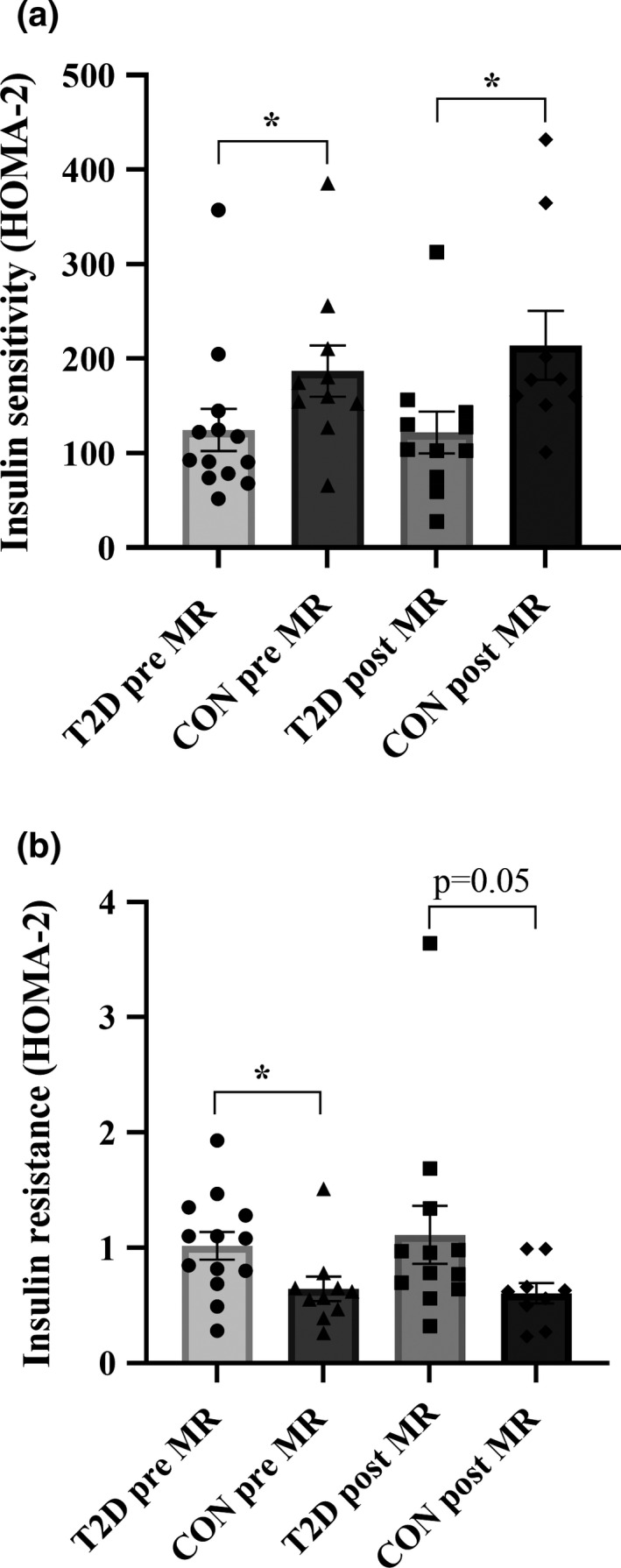
Difference in calculated baseline sensitivity (panel A) and insulin resistance (panel B) by HOMA2 between the individuals with type 2 diabetes and healthy controls before and following mineralocorticoid blockade (MR). CON: healthy controls, T2D: individuals with type 2 diabetes. Pre: prior to MR blockade. Post: following MR blockade. Panel A: pre MR: *p = 0.03, post MR: *p = 0.02. Panel B: pre MR: *p = 0.03

Both before and following the MR blockade, baseline insulin sensitivity was lower in the individuals with type 2 diabetes compared to healthy controls (before: *p* = 0.03, after: *p* = 0.02). Equally, insulin resistance was higher in the individuals with type 2 diabetes compared to healthy controls, before intervention with the MR blockade (*p* = 0.03), but only tended to be higher following intervention (*p* = 0.05), calculated by HOMA2 (Figure 3).

Both before and following the MR blockade, the achieved insulin response increased during clamp stage 1 and 2 compared to baseline, in both the individuals with type 2 diabetes and the healthy controls. Similarly, a decrease in the C‐peptide response was detected at the high dose of infused insulin, and a tendency during the low dose compared to baseline, in the individuals with type 2 diabetes, whereas a decrease occurred during both clamp stages in the healthy controls before MR blockade.

Both before and following the intervention with MR blockade, plasma glucose concentration was higher in the individuals with type 2 diabetes compared to healthy controls at baseline. No difference in the achieved insulin concentration was detected between the individuals with type 2 diabetes and the healthy controls, before the MR blockade. Following the MR blockade, the achieved insulin concentration was lower during clamp stage 1 in the individuals with type 2 diabetes compared to healthy controls (*p* = 0.02). The measured C‐peptide response showed an increase at baseline and during the low dose of infused insulin before MR blockade but only showed an increased at baseline in the individuals with type 2 diabetes compared to healthy controls, following MR blockade.

## 4 DISCUSSION

We aimed to investigate the effect of MR blockade on insulin sensitivity in individuals with type 2 diabetes compared to healthy controls, assessing insulin sensitivity by performance of a two‐stage hyperinsulinemic‐isoglycemic clamp. We additionally estimated baseline insulin sensitivity and calculated insulin resistance by HOMA2. Our results suggest that 8 weeks of MR blockade, does not have a beneficial effect on insulin sensitivity in individuals with type 2 diabetes compared to healthy controls.

### 4.1 MR blockade and insulin sensitivity

During infusion of the high dose of insulin, a decrease in GIR occurred in the individuals with type 2 diabetes, following MR blockade compared to before MR blockade. No change in the achieved insulin concentration or the C‐peptide response was detected during infusion of 50 mU∙m^−2^ min^−1^ in the individuals with type 2 diabetes, following MR blockade compared to before MR blockade. The achieved GIR during the hyperinsulinemic‐isoglycemic clamp method, is a direct measurement of insulin sensitivity, and the detected decrease in GIR could indicate a regression of insulin sensitivity in the individuals with type 2 diabetes, as a consequence of MR blockade. In contrast, an unchanged GIR was present in the healthy controls, suggesting that the present results might reflect the time course and not the MR blockade *per se* (Fonseca, 2009). Even though the included individuals with type 2 diabetes were in pharmacological treatment with no known micro‐ or macrovascular complications in relation to their diabetes, current pharmacological therapies do not abolish the progressive loss of β‐cell function completely (Fonseca, 2009). However, the absence of an additional control group, receiving placebo for comparison, complicate the interpretation of this observation.

Both before and following the MR blockade, a higher HbA1c was present in the individuals with type 2 diabetes compared to the control group (Type 2 diabetes: before: 7.0 ± 3.3%, after: 6.8 ± 3.1%). The levels of HbA1c are predictive of vascular complications in type 2 diabetes (Kirwan et al., 2017). An association between the level of HbA1c and plasma renin has previously been shown, suggesting an increased release in renin with a simultaneous deterioration of the glycemic control (Griffin et al., 2018), as renin is a precursor of aldosterone (Fountain & Lappin, 2020). This suggests that a higher HbA1c, reflecting the present levels of hyperglycemia, could contribute to an increased synthesis of plasma aldosterone, due to the aberrations in glucose metabolism (Huynh et al., 2014). In addition, previous studies have demonstrated an increase in levels of HbA1c in individuals receiving the non‐selective MR antagonist Spironolactone (Yamaji et al., 2010; Zhao et al., 2016), whereas no changes in HbA1c were observed with the selective MR antagonist Finerenone in Phase II studies (Agarwal et al., 2020). The individuals with type 2 diabetes presented with a higher calculated insulin resistance at baseline compared to the control group, and in combination with a higher HbA1c, this could contribute to an increase in the future risk of cardiovascular disease in the individuals with type 2 diabetes, due to their chronic disease.

A decrease in systolic blood pressure was detected at baseline in the control group, whereas a trend toward a decrease was observed in individuals with type 2 diabetes (*p* = 0.05) following the intervention with MR blockade. A larger variance in the change in systolic blood pressure was present in the individuals with type 2 diabetes, which likely explains the difference in response to MR blockade and not insulin *per se*.

### 4.2 MR blockade, insulin levels, and glucose metabolism

During both the low and high dose of infused insulin, plasma insulin levels rose to a high, but physiological plateau (Melmed et al., 2016), compared to baseline during both clamps in both the individuals with type 2 diabetes and the healthy controls. Concordantly, the measured C‐peptide response decreased, as a measure of the endogenous insulin secretion (Leighton et al., 2017). Compared to measurements of insulin, C‐peptide is a more reliable representative of β‐cell function, as C‐peptide is secreted equimolarly to insulin and are not extracted by the liver, making the half‐life of the molecule quite longer (10–30 min vs. 4 min) (Leighton et al., 2017). Thus, the decrease in C‐peptide in response to exogenous insulin reflects a decrease in the endogenous insulin production during the hyperinsulinemic‐isoglycemic clamp and thus, a suppression of the hepatic contribution in glucose metabolism presumably was achieved.

During the low dose of infused insulin, a lower achieved plasma insulin concentration was present in the healthy controls compared to the individuals with type 2 diabetes following intervention. This unexpected difference was not interpreted as a cause of the MR blockade *per se*, as no difference was detected in the achieved levels of plasma insulin in the individual groups both before and after MR blockade. Insulin was infused at an individual rate calculated per BSA on both experimental days. No difference was detected in mean weight and BMI in the individual groups following MR blockade.

Collectively, no beneficial effect of MR blockade on insulin sensitivity was detected in individuals with type 2 diabetes compared to healthy controls. The present data were collected in Caucasians, and as such, it is not known, if these data could be extrapolated to other populations, that is, African Americans. The prevalence of type 2 diabetes is higher among African Americans, where activation of the renin–angiotensin–aldosterone system may play a significant role (Joseph et al., 2018). To the best of our knowledge, MR blockade as a mono‐therapy has not been examined in a population of African Americans, and thus, MR blockade may not reflect the same response in other populations. Nevertheless, the present results are in agreement with previous studies, showing no difference in insulin resistance following treatment with Eplerenone in obese and hypertensive subjects (Adachi et al., 2019), as well as on insulin resistance in metabolic syndrome (Hwang et al., 2015), but to the best of our knowledge, the effect of MR blockade, as a mono‐therapy, has not previously been investigated in individuals with type 2 diabetes.

### 4.3 Limitations

As the present data are a part of a larger project (Finsen et al., 2020), a power analysis was not conducted exclusively for this study. However, it is unlikely that a larger sample size would have altered the outcome, and our findings are additionally supported by previous studies in other populations (Adachi et al., 2019; Hwang et al., 2015), suggesting no effect of MR blockade on insulin sensitivity. The present method represents an isoglycemic state more than an euglycemic during the created hyperinsulinemia. This could influence on the present results, as the levels of plasma glucose concentration were higher in the individuals with type 2 diabetes compared to the control group at baseline, both before and following the intervention with the MR blockade. Fifty percent of the included individuals with type 2 diabetes presented with a BMI between 30 and 32 kg [m^2^]^−1^ and were thus considered obese. Careful evaluation of the individual results suggested that there was no difference in the response between individuals with BMI <30 and ≥30 kg [m^2^]^−1^, and the lack of beneficial effect of the MR blockade due to obesity, are therefore considered unlikely. An inclusion criterium was a sedentary lifestyle with exercise ≤2 h a week, and the performed exercise was not to be high‐intensity training. This assumption was based on the individual subjects own reporting, but not recorded.

## 5 CONCLUSION

The aim of this study was to investigate if MR blockade would have a beneficial effect on insulin sensitivity in individuals with type 2 diabetes. To the best of our knowledge, this is the first study conducted in individuals with type 2 diabetes, examining the effect of MR blockade on insulin sensitivity compared to healthy controls. No beneficial effect of MR blockade was detected on insulin sensitivity in the individuals with type 2 diabetes compared to healthy controls. The present results are supported by previous findings in other patient groups (Adachi et al., 2019; Hwang et al., 2015), but it remains unknown if a longer duration of MR blockade or MR blockade in other stages of T2D, would have a different outcome than the present results. Further studies are, therefore, warranted.

## CONFLICT OF INTEREST

The authors declare no conflict of interest.

## AUTHOR CONTRIBUTIONS

SLHF collected the data, analyzed, and interpreted the data and drafted the manuscript. MRH, JHP, and HFH collected the data, interpreted the data, and revised the manuscript. SPM was responsible for conception and design, collecting the data, analyzing, and interpretation of the data and drafting the manuscript. All authors approved the final version of the manuscript.
